# The genomic prehistory of the Indigenous peoples of Uruguay

**DOI:** 10.1093/pnasnexus/pgac047

**Published:** 2022-04-21

**Authors:** John Lindo, Rosseirys De La Rosa, Andre L C d Santos, Mónica Sans, Michael DeGiorgio, Gonzalo Figueiro

**Affiliations:** Department of Anthropology, Emory University, Atlanta, GA 30322, USA; Department of Anthropology, Emory University, Atlanta, GA 30322, USA; Department of Archeology, Federal University of Pernambuco, Recife, Brazil; Department of Electrical Engineering and Computer Science, Florida Atlantic University, Boca Raton, FL 33431, USA; Departamento de Antropología Biológica, Facultad de Humanidades y Ciencias de la Educación, Universidad de la República, Montevideo, Uruguay; Department of Electrical Engineering and Computer Science, Florida Atlantic University, Boca Raton, FL 33431, USA; Departamento de Antropología Biológica, Facultad de Humanidades y Ciencias de la Educación, Universidad de la República, Montevideo, Uruguay

## Abstract

The prehistory of the people of Uruguay is greatly complicated by the dramatic and severe effects of European contact, as with most of the Americas. After the series of military campaigns that exterminated the last remnants of nomadic peoples, Uruguayan official history masked and diluted the former Indigenous ethnic diversity into the narrative of a singular people that all but died out. Here, we present the first whole genome sequences of the Indigenous people of the region before the arrival of Europeans, from an archaeological site in eastern Uruguay that dates from 2,000 years before present. We find a surprising connection to ancient individuals from Panama and eastern Brazil, but not to modern Amazonians. This result may be indicative of a migration route into South America that may have occurred along the Atlantic coast. We also find a distinct ancestry previously undetected in South America. Though this work begins to piece together some of the demographic nuance of the region, the sequencing of ancient individuals from across Uruguay is needed to better understand the ancient prehistory and genetic diversity that existed before European contact, thereby helping to rebuild the history of the Indigenous population of what is now Uruguay.

Significance Statement The Indigenous people of Uruguay suffered devastating consequences after the arrival of Europeans, to the point of near extinction. Here we present the first ancient genomes from the region, before European contact, and elucidate a nuanced population history exhibiting a migration route along the Atlantic Coast of South America and a putative new ancestry, previously undetected in the continent.

## Introduction

Historically, Uruguayan identity has been marked by the extermination of the Indigenous populations of the region at the time of European contact in the 16th century and up until the 19th century. The exterminations were carried out through a series of military campaigns, culminating in the massacre at Salsipuedes creek in 1831 (1). The target of the Salsipuedes campaign was the ethnic group known as the Charrúa (2), which at the time was the term employed for the remnants of various hunter–gatherer groups in the recently independent territory of Uruguay. Subsequently, it was held that in sharp contrast to all other South American countries, Uruguay lacked Indigenous populations—an idea still widely accepted. However, population genetic studies beginning in the 1980s proposed a significant Indigenous contribution (e.g. (2)), sparking an increased interest in the Native genetic background of the population.

In 1989, the Charrúa reemerged in Uruguay. Beginning with the founding of the association of descendants of the Charrúa people (*Asociación de Descendientes de la Nación Charrúa*—ADENCH), an evolution in the identity of the descendants of Native peoples took place. In 2005, the various Indigenous collectives integrated in the *Consejo de la Nación Charrúa* (CONACHA) and declared themselves Charrúas, later initiating diverse political actions toward the formalization of their status (3).

However, this change in perspective is not directly related to the advances in genetic studies, and though the Indigenous genetic background of the Uruguayan population has been firmly established (see (4) for a review), its ethnic basis is far from resolved. In relation to the extermination of the Charrúa, it must be noted that while the military campaigns were directed at the Charrúa, Guaraní Indians continued entering the territory until 1828, when General José F. Rivera brought approximately 8,000 Guaraní from the former Jesuit Missions. These numbers, in contrast with the numbers of Charrúa tallied by the hundreds in 1831 (1) has led to the suggestion that the major Indigenous contribution to the Uruguayan population would have been Guaraní (5).

The link between past and present populations turns vaguer as we retreat to the time of European contact. The data and nomenclatures regarding the Indigenous ethnic groups existing in the present territory of Uruguay in the 16th century are varied, though there is a certain consensus regarding the presence of two major groups. On the one hand, there is the so-called Charrúa macro-ethnic group, which includes the Guenoas, Bohanes, Yaros, and the Charrúas themselves. On the other hand, the Guaraní, initially observed in the areas near the great rivers, would have arrived in the present Uruguayan territory shortly before the arrival of the Europeans (16). Apart from this, canoeists and horticulturists of the Uruguay River are also reported, by the name of Chanás (1). The Chanás have an unclear link with the Charrúa and their culture underwent profound changes by the beginning of the 18th century (17). Further back, caution has been exercised regarding possible associations between the ethnic groups recorded in the first chronicles and those recorded in the archaeological record, as is the case of the mounds of Eastern Uruguay, which are presented here.

The research presented here aims to elucidate the genomic prehistory of the Indigenous people of Uruguay by presenting low-coverage whole genomes from the CH2D01-A archeological site in Rocha, Uruguay, which date from ∼1,450 to ∼668 years before present (BP; Table 1; Figure S1, Supplementary Material). This represents the first ancient genomic DNA from the region and presents a starting point to examine the evolutionary history of the Indigenous people of Uruguay and their diversity from a genomic perspective.

**Table 1.** Ancient samples sequenced in this study from the archaeological site CH2D01-A in Rocha, Uruguay. *Radiocarbon dating was conducted at the University of Arizona AMS Laboratory. ^†^Sex was determined via the aligned sequences with SEXCMD (6). ^‡^Contamination was estimated from mtDNA alignments using Schmutzi (7) and from the X chromosome using ANGSD (8). Sample CH13 did not have enough informative sites for sex determination.

			mtDNA haplogroup	Contamination^‡^		Endogenous DNA (%)	Mean sequencing depth	Genome coverage
Sample	^14^C age*	Sex^†^		mtDNA X	Chromosome			
CH13	668 ± 22 BP	–	C1d3	.01, 95% CI [0 to 0.02%]	–	3.4	10.7	0.137
CH19B	1450 ± 70 BP	XX	C1c	.05, 95% CI [.04 to 0.06%]	0.034909 SE: 3.287883e-03	5.1	7.14	0.344

## Results

**Fig. 1.**
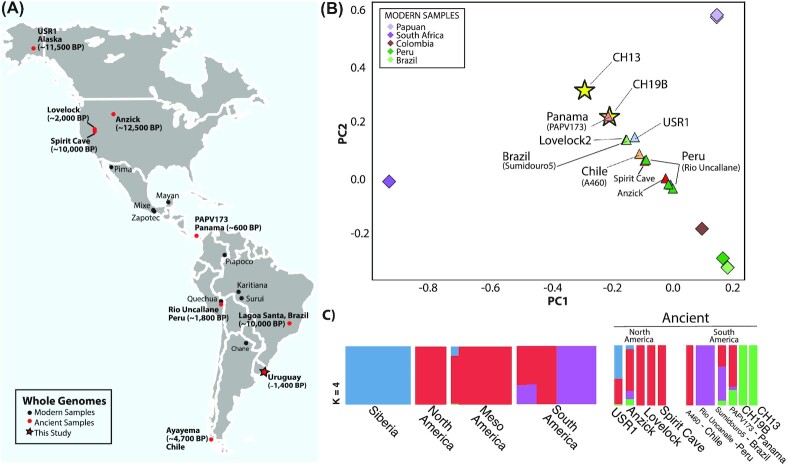
Assessing the genetic affinity of ancient Uruguay with the Americas. (A) Map of ancient and modern whole genomes used in this study (9–14). (B) Principal component analysis projection of the ancient Uruguay samples on to the first two principal components (PCs). Diamonds indicate contemporary samples and triangles indicate ancient samples. (C) Ancestry clusters generated with ADMIXTURE (15) of modern and ancient genomes from the Americas at *K* = 4 clusters, which was chosen through cross-validation value.

To assess the relationship of the ancient Uruguay samples with global and regional populations, both ancient and modern, we merged the dataset with samples from the Simons Genomes Diversity Project (9) and ancient whole genomes from the Americas (10–14). To prevent a batch effect from the method used to call the ancient Uruguay genotypes, the ancient reference BAM files were also called with the ancient DNA caller ARIADNA (18) (see Methods). To maximize the number of overlapping sites for the various population genetic analyses, only whole genomes were chosen for comparison (Fig. 1A).

### Mitochondrial DNA

The mitochondrial genome of CH13 carries all diagnostic mutations of haplogroup C1d, namely 194T and 16051G,  including the additional mutations 12378T, 16140C, and 16288C, which place it within subhaplogroup C1d3 (Figure S3, Supplementary Material). This subhaplogroup had its origin approximately 9,000 years ago and apparently evolved entirely in what is now Uruguay, and is found also in CH20, an individual uncovered in the same site but between 700 and 1,000 years older (20). The mtDNA of CH19B carries mutations 1888A and 15930A, diagnostic of haplogroup C1c, but lacks further mutations associated with registered subhaplogroups (C1c1 through C1c8). Furthermore, CH19B carries 606G and a deletion in position 7,471, neither of which have been found in published mitochondrial sequences. The lineage represented by CH19B might very well be extinct.

### Genomic analyses

We performed a principal component analysis to better understand the relationship of the ancient Uruguay individuals with other ancient individuals from the Americas. C/T and G/A transitions were removed from the dataset for all analyses, except where otherwise noted, to guard against the most common forms of postmortem DNA damage (21) and to prevent false affinities among the ancient samples. Because the Uruguay ancient individuals are low coverage compared to the modern and ancient high coverage samples in the reference panel (9–12, 14, 22), we projected the ancient genomes onto the top two principal components (PCs) identified from the modern samples using SmartPCA (23). Interestingly, the contemporary ancient samples from Uruguay (CH19B, ∼1,400 BP) and Panama (PAPV173 (14), ∼1,400 BP) show a strong affinity on the first two PCs (Fig. 1B), with the more recent Uruguay sample (CH13, ∼600 BP) showing a more distant relationship. To further elucidate the relationship among the ancient Uruguay individuals and the Americas, we performed an ADMIXTURE-based cluster analysis, with *K* = 4 clusters showing the best cross-validation value (Fig. 1C). The Uruguay ancient samples exhibit a green ancestry cluster that is shared with USR1 (11) (Alaska, ∼11,500 BP) and Anzick-1 (10) (Montana, ∼12,500 BP). In relation to the South America, the green cluster is shared with Sumidouro5 (12) (Brazil, ∼10,000 BP) and PAP173 (14) (Panama, ∼1,400 BP), which shows the largest shared fraction. With regard to the modern samples in the reference panel, the green cluster is apparent in a Mayan individual (9) but is not observed in other populations.

**Fig. 2.**
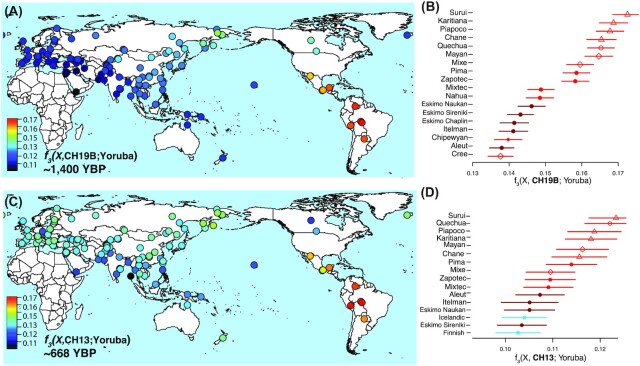
Outgroup ƒ_3_ statistics. Left (A) and (C): heat maps represent the outgroup ƒ_3_ statistics quantifying the amount of shared genetic drift between the ancient Uruguay individuals and each of the contemporary populations from the Simons Genome Diversity Project (19) since their divergence with the African Yoruban population. Right (B) and (D): ranked ƒ_3_ statistics showing the greatest affinity of the ancient Uruguay with respect to Indigenous populations of the Americas.

To further examine the relationship of the ancient individuals from Uruguay with global populations, we utilized outgroup ƒ_3_ statistics to assess the shared genetic ancestry with the modern individuals from Simons (9). Outgroup ƒ_3_ statistics of the worldwide dataset demonstrate that both ancient Uruguay individuals display greater affinity with Indigenous groups from South America than with other populations (Fig. 2A and C). Ranked outgroup ƒ_3_ statistics suggest that both ancient individuals from the two time periods (CH19B: ∼1,450 BP and CH13: ∼668 BP) tend to share the greatest affinity with Brazilian living groups, the Surui and Karitiana (Fig. 2B). However, we do not detect a shared Austronesian signal in either ancient individual, which may suggest a more nuanced relationship with the Amazonian populations.

**Fig. 3.**
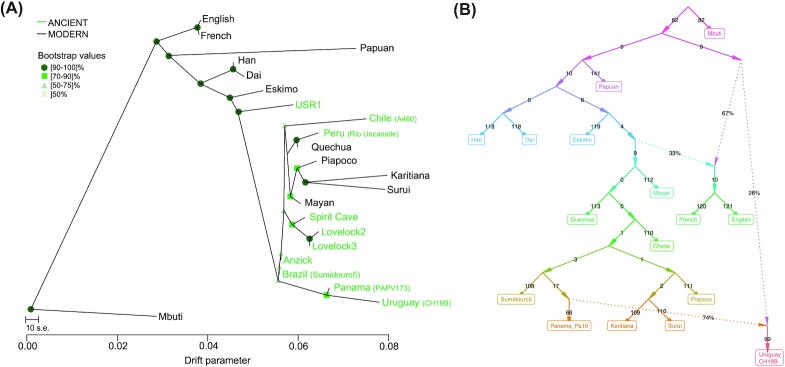
Maximum likelihood Tree and qpGraph. (A) Maximum likelihood trees generated by TreeMix (24) using whole-genome sequencing data from the Simons Genome Diversity Project (9). The tree shows a connection between ancient samples from eastern Brazil, Panama, and Uruguay. (B) The qpGraph best fitting model with two migration events. The populations included derive from the Simons (9) and shows a deep ancestral event in the direction of the ancient Uruguay sample (CH19B), along with a migration signal associated with ancient Panama. Branch of length zero should be interpreted as no evidence of drift separating the splits. The model depicted had a score of 319.42, utilizing 140,782 SNPs.

To explore the relationships between the ancient Uruguay individuals and the Americas, we utilized maximum likelihood trees inferred with TreeMix (24). The ∼10,000 BP ancient sample from Brazil, Sumidouro5, falls basal to both ancient Panama and ancient Uruguay (Fig. 3A). With two migrations, gene flow is detected between both ancient individuals from Uruguay to Sumidouro5 (Figure S5, Supplementary Material). This could be indicative of a relationship between the samples that may be due to a common migration route or shared ancestry, which might be distinct from the migrations/ancestry that led to the modern Amazonian populations, the Surui and the Karitiana. The geographic positioning of the samples may also support this, as Sumidouro5 is from an archeological site on the eastern coast of Brazil and the Amazonian populations are located in the western part of the country (Fig. 1A). Though we are careful not to claim definitive relationships among the ancient and modern samples, the tree does seemingly correctly position the ancient individuals from the highlands of Peru (Rio Uncanalle (13)) with modern-day individuals from the same region, the Quechua (9). Furthermore, the ∼11,500-y-old USR1 (11), a terminal individual from Alaska, is placed as an outgroup to all populations from the Americas, which lends additional validation of the tree structure.

We also tested the relationship of the 1,400-y-old ancient Uruguay individual with modern South American populations using qpGraph (25), which employs the set of ƒ_2_ statistics across numerous population pairs to estimate the topology of an admixture graph. We found that the model fit best with two migration events, incorporating the individuals from key populations of the Simons Genome Diversity Project (9) (Fig. 3B). The topology of the graph also suggests that ancestry of the ancient Uruguay sample is deriving from two sources: a deep ancestral source and a source that led to the Karitiana and Surui of Brazil. Both the maximum likelihood tree and the qpGraph show a more complicated picture than what was shown with the outgroup ƒ_3_ statistic, whereby the Amazonian populations share a more distant connection to the ancient Uruguay individuals. This connection may relate to a more general ancestry signal from South America, rather than a direct one, and may be a consequence of different migrations upon entry into the continent. In contrast, we see a connection with ancient Brazil, Panama, and Uruguay on the maximum likelihood tree, where they form their own branch (Fig. 3A). The qpGraph shows a connection between the ancient Panama sample and the oldest Uruguay individual, demonstrating a migration event, as defined by the qpGraph, between the two (Fig. 3B). Taken together, it is possible that the connections are reflective of migration routes that occurred along the Atlantic coast of South America.

We also note a deep ancestral component contributing to the ancient sample form Uruguay (Fig. 3B), which combined with the ancestry cluster results (Fig. 1C), may represent a previously undetected ancestry in South America. This distinct ancestry may also corelate with the mitochondrial lineage of CH19B, which might be extinct. However, this ancestry seems present in both Anzick-1 (Montana) and USR1 (Alaska) (Fig. 1C, *green ancestral component*), both of which are considered ancestral to the Indigenous populations of the Americas. We also note that the deep ancestral lineage is present in the more recent CH13 (Figures S1 and S2, Supplementary Material).

## Discussion

**Fig. 4.**
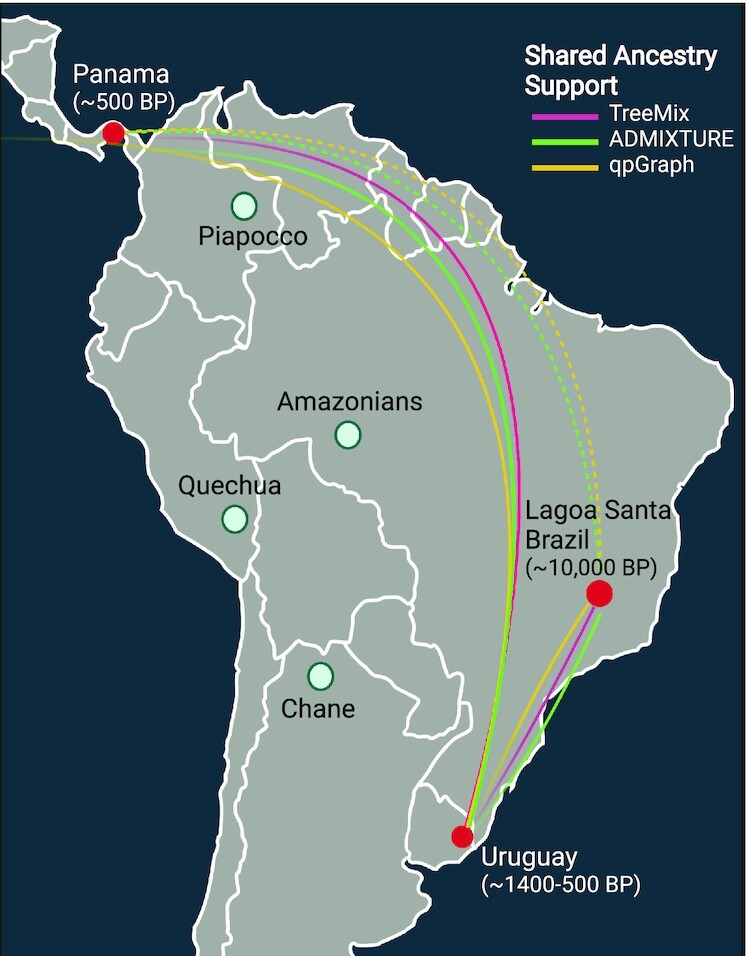
Evidence of shared ancestry with the ancient individuals from Uruguay.

Here, we start to elucidate the origins of the Indigenous people of Uruguay. We find that the ancestral population of the ancient Uruguay individuals may have derived from a migration that stemmed closer to the Atlantic coast. This is evidenced by the affinity to the Sumidouro5 ancient individual, found closer to the southeastern Atlantic coast of Brazil (Fig. 4), and is supported by paleoenvironmental and chronological data (26). This migration may be different than those that led to modern Amazonian Indigenous populations from Brazil, given that these populations repeatedly form a distinct group on our various analyses. We also find an unexpected, shared ancestry to an ancient Panama sample, some 5,000 km away, which could possibly point to a shared migration route from North America or possibly migrations back into Mesoamerica.

While we begin to unravel the relationship of the Indigenous people of Uruguay on a continental level, in addition to the potential discovery of a distinct ancestral component in South America, it is also important to point out the need for ancient DNA from other archaeological sites from across Uruguay, especially those close to the time of European contact. Such samples will help to better capture the genetic diversity of the Indigenous people that existed upon contact with the Spanish in the 16th Century, and by extension, better understand the diversity of Indigenous groups that existed. In doing so, future DNA studies could assist living people in Uruguay to potentially identify Indigenous ancestry that is not limited to the “Charrúa.” Furthermore, the analysis of the ancient DNA data from CH2D01-A, in the light of Indigenous ancestry found in contemporary populations from Uruguay (27) will help to unravel the diverse components of the genetic continuity between prehistoric and living populations first glimpsed through mtDNA data.

## Methods

### Archaeology and samples

Research work developed in the Department of Rocha, Uruguay, since the 1980s has focused on the study of monticular structures (*cerritos de indios*), which show complex socio-cultural processes involving local populations from more than 5000 years ago until the 17th century. With diameters between 30 and 40 m and variable heights that can reach 7 m, the mounds show a planned action that pursued the conditioning of specific places in geographic space. The archaeological record of the area corresponds to hunter–gatherer groups with the presence of horticulture. Within these constructions, the presence of human burials is frequent, with a chronological range from 1,610 ± 46 BP (28) to 220 ± 50 BP (29). The skeletal remains recovered from the *cerritos* have been the subject of several studies including genetic relationships through ancient mitochondrial DNA. In particular, the analysis of human remains from site CH2D01-A (28) showed that two of them belonged to subhaplogroup C1d3, a variant restricted to Uruguay and found in the current population. Subsequent analyses of complete mitochondrial genomes (20) established the variability and temporal depth of the lineage, but the details of the relationship of the populations buried in the *cerritos* with historical and prehistoric populations persist.

The two samples presented here derive from site CH2D01, which is a group of two mounds (A and B). The radiocarbon dating place the occupation of mound A between 2,000 BP and the period of European contact. The mound is approximately 1.20 m high with a diameter of 35 m and is presumed to be an area of differentiated activity within a larger site of about 20,000 m^2^. The archaeological materials recovered from mound A do not show clear spatial arrangements and were interpreted as “displaced primary contexts”: materials that were carried along with the sediments that make up the mound from the places where the activity was carried out. Implicit in this interpretation is the intentional character of the mound construction. Though there is ongoing debate about the exact mechanism of the formation of the *cerritos*, there have been no subsequent interpretations about site CH2D01. In excavation IA, a 25 m^2^ excavation carried out in the center of mound A, several bone assemblages representing the primary and secondary burials of at least 21 individuals were recovered.

### Ancient DNA and sequencing

The ancient tooth samples were extracted, and sequencing libraries were constructed at the Lindo Ancient DNA laboratory at Emory University using the Dabney protocol (30). Libraries were prepared with the NEB Ultra II DNA Library Prep for Illumina, with modifications for ancient DNA, which including quartering the reagents, the utilization of 1:20 adaptor dilution, and 1.5 ul of premixed NEB indexes. Samples were preliminarily screened for endogenous DNA on the Illumina iSeq 100 at the Lindo Lab, with libraries that were not treated with the USER enzymes. Samples that were selected for deep sequencing on the NovaSeq 6000 at Dante Labs (L'Aquila, Italy), included libraries treated with the USER enzyme to help compensate for DNA damage.

The ancient raw sequences were trimmed for Illumina adapters using AdapterRemoval2 (31) and aligned to the hg19 human reference sequence using the BWA mem algorithm (32), which has been shown to increase accuracy with ancient DNA mapping over the *aln* algorithm (33). The alignments from the shotgun sequences that were not treated with the USER enzyme were used to validate their ancient authenticity with MapDamage2 (34). Both ancient individuals showed deamination patterns consistent with ancient DNA (Figure S4, Supplementary Material).

Genotypes were called from both ancient individuals with the ancient DNA caller ARIADNA, which uses a machine learning method to overcome issues with DNA damage and contamination (18). The resulting VCF was further filtered to remove genotype calls with allele counts below 3. Because CH19B showed a relatively high contamination rate, we further filtered the associated VCF using RFMix (35) to identify and remove sites that showed a high probability of deriving from Europeans. The VCFs were then merged with modern and ancient samples from the Americas with bcftools (36).

### SmartPCA

We conducted principal component analysis using the “smartpca” program from the EIGENSOFT v7.2.1 package. PCs were estimated with the “poplistname” option and using representative individuals from present-day Native American and Indigenous South African and Papuan populations from Simons Genome Diversity Panel (9). The ancient individuals were then projected onto the computed PCs with the “lsqproject: YES” option. No outliers were excluded, LD pruning was not performed, and the analysis was based on 4,978,400 loci.

### Assessment of population structure using ADMIXTURE

We started with the identical filtered dataset of called genotypes described above. We further pruned the dataset by removing sites in strong linkage disequilibrium (${r^2} > 0.1$) using PLINK (37). The program *ADMIXTURE* was used to assess global ancestry of the ancient and present-day samples from this study. We computed cluster membership for *K* = 2 through *K* = 15, running 10 replicates for each *K* value while generating pseudo-random seed with the -s option. The replicate with the best likelihood was then chosen for each *K*. We found the lowest cross-validation value to be at *K =* 4. The PONG (38) program was used to visualize the admixture plots.

### Outgroup ƒ_3_ analysis

We applied the *qp3Pop* module of *ADMIXTOOLS* (25) to compute *f*_3_ statistics with the target population as the African Yoruban population and the two reference populations set as one of the ancient Uruguay samples (CH13 or CH19B) and the other as one of the non-African populations from the Simons Genome Diversity Project (9). For this analysis, we retained C/T and G/A transitions, as the CH13 and CH19B ancient samples have been treated with uracil–DNA glycosylase to guard against this form of DNA damage. For sample CH13, a minimum of 46,982 and a maximum of 51,289 SNPs were used. For sample CH19B, a minimum of 122,882 and a maximum of 132,499 SNPs were used.

### TreeMix analysis

We started with the identical filtered dataset of called genotypes described above. *TreeMix* was applied to the dataset to generate maximum likelihood trees and admixture graphs from allele frequency data. The Mbuti from the Simons dataset was used to root the tree (with the *–root* option). We accounted for linkage disequilibrium by grouping *M* adjacent SNPs (with the *–k* option), and we chose *M* such that a dataset with *L* sites will have approximately $L/M \approx 20,000\ $ independent sites. At the end of the analysis (i.e. number of migrations) we performed a global rearrangement (with the *–global* option). We considered admixture scenarios with $m\ = \ 0\ $ and $m\ = \ 3$ migration events. Each migration scenario was run with 500 bootstrap replicates, and the replicates were used to determine the confidence of each node.

### qpGraph analysis

We employed the *ADMIXTOOLS2* (https://uqrmaie1.github.io/admixtools/index.html, *ADMIXTOOLS2* is currently under preparation) R package to perform *qpGraph* (25) estimation. We extracted *f*_2_ statistics between population pairs using a two megabase SNP block size. We considered scenarios with $M \in \{ {0,1,2,3,4,5,6} \}$ migration events, with graph searches initiated by a random graph and Mbuti population set as the outgroup, stopping the search after 100 generations. If the best graph with *M* events did not have a better score than those with fewer events, then the graph search was rerun. The best-fit model of two migration events was chosen by assessing statistical differences between model score distributions computed from 1,000 bootstrap replicates of *f*_2_ blocks. For sample CH19B, 140,782 SNPs were used. For sample CH13, 54,502 were used.

## Supplementary Material

pgac047_Supplemental_File

## Data Availability

The sequence data, in BAM format, for CH13 and CH19B are available at the European Nucleotide Archive under the accession number PRJEB48360.
