# Supplementary material for: The genomic prehistory of the Indigenous peoples of Uruguay
Source: PNAS Nexus. 2022 Apr 21;1(2):pgac047. doi: 10.1093/pnasnexus/pgac047 (PMC9802099; doi:10.1093/pnasnexus/pgac047)
Supplement: pgac047_Supplemental_File [file pgac047_supplemental_file.docx]

**The Genomic Prehistory of the Indigenous Peoples of Uruguay**

John Lindo^1^*, Rosseirys De La Rosa^1^, Andre Luiz Campelo dos Santos^2,3^, Mónica Sans^4^, Michael DeGiorgio^3^, Gonzalo Figueiro^4*^

**Supplement**

**S1. Archaeology context**

*Cerritos de indios*

Research work developed in the Department of Rocha, Uruguay, since the 1980s has focused on the study of monticular structures (*cerritos de indios*), which show complex socio-cultural processes involving local populations from more than 5000 years ago until the 17th century. With diameters between 30 and 40 meters (m) and variable heights that can reach 7 m, the mounds show a planned action that pursued the conditioning of specific places in geographic space. The archaeological record of the area corresponds to hunter-gatherer groups with the presence of horticulture; the function of the *cerritos* is, however, subject to debate, having been attributed habitation, ritual, and monumental functions (for general reviews, *see* (1–3)).

Within these constructions, the presence of human burials is frequent (for a detailed discussion of burial modes, see (4), with a chronological range from 1610±46 BP (5) to 220±50 BP (3). The skeletal remains recovered from the *cerritos* have been the subject of several studies including genetic relationships through ancient mitochondrial DNA. In particular, the analysis of human remains from site CH2D01-A (5) showed that two of them belonged to subhaplogroup C1d3, a variant restricted to Uruguay and found in the current population. It is also found in the remains of Vaimaca Peru, the only remains of a Charrúa Indian whose mtDNA was analyzed through the sequencing of hypervariable segment I (5). Subsequent analyses of complete mitochondrial genomes (6) allowed establishing the variability and temporal depth of the lineage, but the details of the relationship of the populations buried in the *cerritos* with historical and prehistoric populations persist.

**S2. Sequencing statistics and comparative populations**

| **Sample ID** | **Total reads** | **% Duplication** | **Mapped unique** | **Mean**  **Read Length** | **Mean mtDNA**  **Read Depth** |
| --- | --- | --- | --- | --- | --- |
| CH13 | 771932837 | 0.375709 | 235006673 | 67 | 207.4 |
| CH19B | 2362704934 | 0.393612 | 349058159 | 69 | 44.5 |

**Table S1. Sequencing statistics for the two ancient genomes from Uruguay.**

| **Sample ID** | **Location** | **Mean Sequence Depth** | **Radiocarbon Dates (Ancient Samples)** |
| --- | --- | --- | --- |
| **Ancient Samples** | | | |
| Ayayema (A460) | Patagonia, Chile | 10.6x | 5.1 kya |
| Spirit Cave (AHUR_206) | Nevada, USA | 18x | 10.7 kya |
| Lovelock2 | Nevada, USA | 15.1x | 1.9 kya |
| Lovelock3 | Nevada, USA | 18.7x | 0.7 kya |
| Sumidouro5 | Lagoa Santa, Brazil | 15.1x | >10 kya |
| USR1 | Alaska, USA | 17x | 11.5 kya |
| Anzick1 (Clovis) | Montana, USA | 14.4x | 12.8 kya |
| Panama (PAPV173) | Coco del Mar, Panama | 0.58x | 0.5 kya |
| IL2 (Rio Uncallane) | Ilave, Peru | 6.5x | ~1.8 kya |
| IL3 (Rio Uncallane) | Ilave, Peru | 5.9x | 1.8 kya |
| IL7 (Rio Uncallane) | Ilave, Peru | 5.6x | 1.8 kya |
| **Contemporary Samples (Simons Genomes Diversity Project)** | | | |
| S_Pima-1 | Mexico | 43.03x |  |
| S_Pima-2 | Mexico | 37.84x |  |
| S_Mixtec-1 | Mexico | 44.48x |  |
| S_Mixtec-2 | Mexico | 46.91x |  |
| B_Mixe-1 | Mexico | 40.13x |  |
| S_Mixe-2 | Mexico | 46.94x |  |
| S_Mixe-3 | Mexico | 46.66x |  |
| S_Zapotec-1 | Mexico | 45.75x |  |
| S_Zapotec-2 | Mexico | 39.23x |  |
| S_Mayan-1 | Mexico | 37.77x |  |
| S_Mayan-2 | Mexico | 45.13x |  |
| S_Piapoco-1 | Colombia | 40.88x |  |
| S_Piapoco-2 | Colombia | 41.01x |  |
| S_Quechua-1 | Peru | 42.04x |  |
| S_Quechua-2 | Peru | 44.82x |  |
| S_Quechua-3 | Peru | 59.78x |  |
| S_Karitiana_1 | Brazil | 44.93x |  |
| S_Karitiana_2 | Brazil | 39.49x |  |
| B_Karitiana_3 | Brazil | 37.39x |  |
| S_Surui_1 | Brazil | 41.50x |  |
| S_Surui_2 | Brazil | 35.63x |  |

**Table S2. Information regarding comparative whole genomes used in this study.**

**S3. qpGraphs with individual CH13 from Uruguay.**

**Figure S1.**  The qpGraph best fitting model with two migrations the featuring ancient Uruguay sample CH13. The contemporary populations included derive from the Simons (7) and shows a deep ancestral event in the direction of the ancient Uruguay sample (CH13), along with a migration signal associated with the ancient Panama population. Branch of length zero should be interpreted as no evidence of drift separating the splits. The model depicted had a score of 217.08, utilizing 54,502 SNPs.

**A) B)**

**Figure S2.** Residual *f*_2_ matrix for the qpGraphs with **(**A) CH19B and (B) CH13.

**S4. Mitochondrial Analyses of CH13**

**
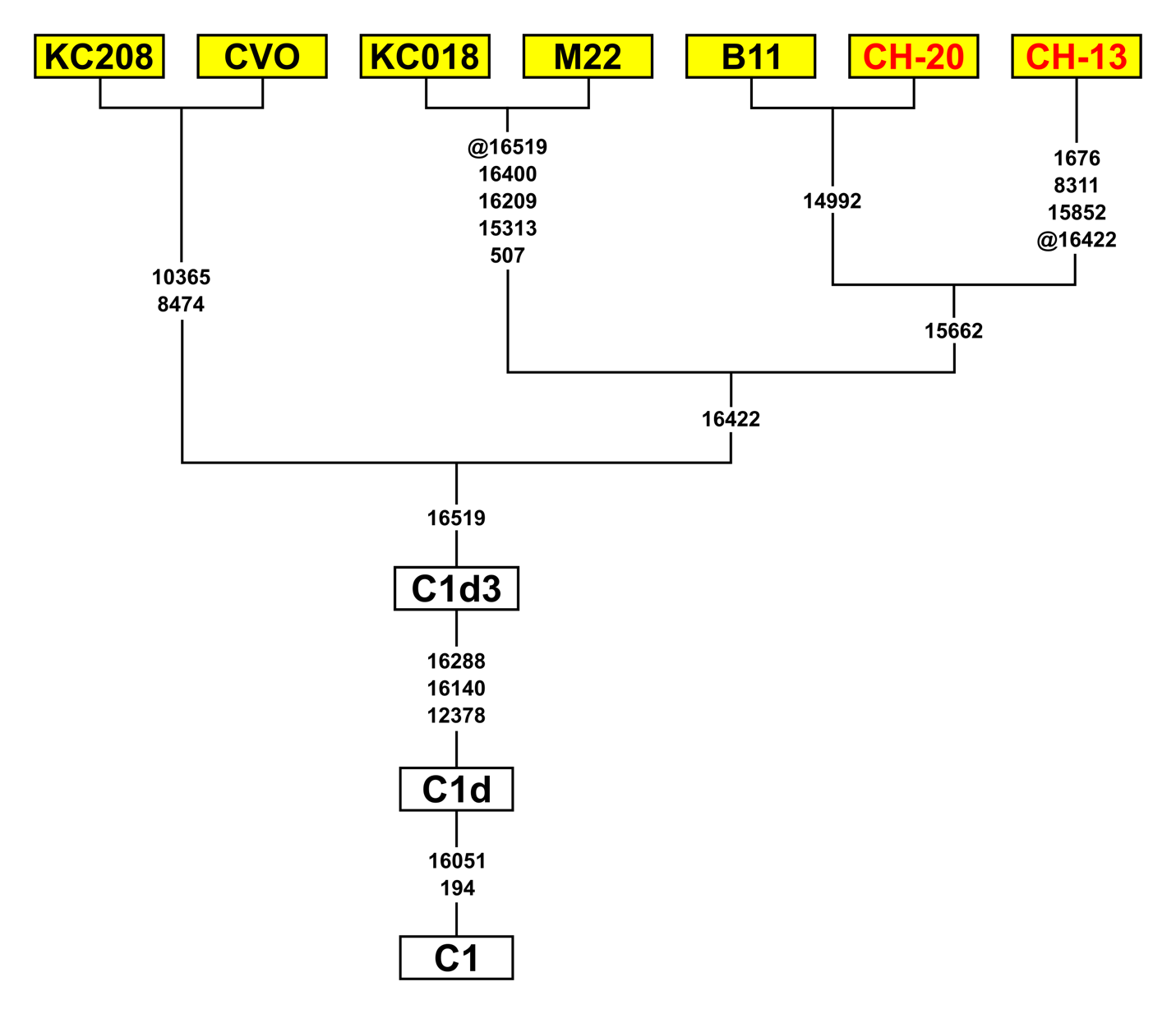
**

**Figure S3. Proposed phylogenetic relationship between CH13** **and published C1d3 mitochondrial genomes.** Labels in red are ancient samples from CH2D01-A. References for the sequences: B11 –Sans et al. (5); CH-20, KC018, KC208, and M22 – Sans et al. (6); CVO (8); CH13 – this study.

**S4. Deamination Patterns**

**Figure S4.** DNA damage patterns for the two ancient samples from Uruguay. Libraries used to detect the deamination patterns were not treated with Uracil DNA glycosylase, though downstream analyses were based on libraries for which the samples were treated**.**

**S5. TreeMix with migrations**

**A) B)**

**Figure S5.** Maximum likelihood graphs generated by TreeMix with two migration events for CH19B (A) and CH13 (B). 20 iterations were performed for each, choosing the best likelihood, and using 404,488 sites for CH19B and 272,121 sites for CH13 analyses.

**References**

1. J. M. L. Mazz, Las estructuras tumulares (Cerritos) del litoral Atlántico Uruguayo. *Latin American Antiquity* 12, 231–255 (2001).

2. J. Iriarte, *et al.*, Evidence for cultivar adoption and emerging complexity during the mid-Holocene in the La Plata basin. *Nature* 432, 614–617 (2004).

3. R. B. Boksar, Montículos de la Cuenca de la Laguna Merín: Tiempo, Espacio y Sociedad. *Lat Am Antiq* 17, 511–540 (2006).

4. G. Figueiro, “Bioarqueología En El Uruguay: Situación Actual y Perspectivas Futuras” in *Avances Recientes En La Bioarqueología Latinoamericana*, L. Luna, C. Aranda, J. Suby, Eds. (Grupo de Investigación en Bioarqueología, 2014), pp. 47–68.

5. M. Sans, G. Figueiro, P. C. Hidalgo, A New Mitochondrial C1 Lineage from the Prehistory of Uruguay: Population Genocide, Ethnocide, and Continuity. *Hum Biol* 84, 287–305 (2012).

6. M. Sans, *et al.*, A South American Prehistoric Mitogenome: Context, Continuity, and the Origin of Haplogroup C1d. *PloS one* 10, e0141808 (2015).

7. S. Mallick, *et al.*, The Simons Genome Diversity Project: 300 genomes from 142 diverse populations. *Nature* 538, 201–206 (2016).

8. G. Figueiro, *et al.*, Filogeografía de mitogenomas indígenas completos del Uruguay. *Revista Argentina de Antropología Biológica, In press,* (2021).
